# A longitudinal study of prediagnostic metabolic biomarkers and the risk of molecular subtypes of colorectal cancer

**DOI:** 10.1038/s41598-020-62129-1

**Published:** 2020-03-24

**Authors:** Robin Myte, Sophia Harlid, Anneli Sundkvist, Björn Gylling, Jenny Häggström, Carl Zingmark, Anna Löfgren Burström, Richard Palmqvist, Bethany Van Guelpen

**Affiliations:** 1 Department of Radiation Sciences, Oncology, Umeå University, Umeå, Sweden; 2 Department of Medical Biosciences, Pathology, Umeå University, Umeå, Sweden; 3 Department of Statistics, Umeå School of Business, Economics, and Statistics, Umeå University, Umeå, Sweden; 4 Wallenberg Centre for Molecular Medicine, Umeå University, Umeå, Sweden

**Keywords:** Cancer epidemiology, Cancer prevention

## Abstract

Body fatness increases the risk of colorectal cancer (CRC). Insulin resistance and altered adipokines are potential mechanisms, but previous biomarker studies have been inconsistent. Intertumoral heterogeneity might provide an explanation. We investigated insulin, C-peptide, adiponectin, and leptin in relation to CRC molecular subtypes using a nested case-control design (1010 cases, 1010 matched controls, median 12.3 years from baseline to CRC diagnosis) from the population-based Northern Sweden Health and Disease Study. Repeated samples were available from 518 participants. Risks of CRC and subtypes, defined by tumor *BRAF* and *KRAS* mutations and microsatellite instability (MSI) status, were estimated using conditional logistic regression and linear mixed models. Higher C-peptide and lower adiponectin were associated with increased CRC risk (odds ratios per standard deviation increase (95% CI): 1.11 (1.01, 1.23) and 0.91 (0.83, 1.00), respectively), though weakened when adjusted for body mass index. Insulin and leptin were not associated with CRC risk. Within-individual time trajectories were similar in cases and controls, and no subtype-specific relationships were identified (all P_heterogeneity_ > 0.1). Adiponectin was weakly inversely associated with the risk of *KRAS*-mutated (P = 0.08) but not *BRAF*-mutated or *KRAS/BRAF*-wildtype CRC, consistent with the one previous study. These findings contribute to an increased understanding of the complex role of body size in CRC.

## Introduction

Body fatness increases the risk of colorectal cancer (CRC)^1,2^. Mendelian randomization studies have consistently inferred the association as causal^3,4^, but the underlying biology is not fully understood. Putative mechanisms include insulin resistance and changes in circulating adipokines – adipocyte-derived cytokines and growth factors, chronic low-grade inflammation, and an altered gut microbiome^5^.

A role for insulin resistance and adipokines in CRC development is biologically plausible^5^. In experimental studies, insulin resistance has been related to increased inflammation and cell proliferation^6^. Adiponectin has been shown to suppress tumor growth by inhibiting cell proliferation, adhesion, and invasion, or by increasing tumor-infiltrating immune cells^7^, and leptin may promote proliferation and angiogenesis or inhibit apoptosis^8^. Results from epidemiological studies of circulating biomarkers related to these mechanisms and CRC risk have been somewhat inconsistent. For insulin and C-peptide (markers of insulin resistance), higher circulating levels have been associated with an increased CRC risk, with moderate heterogeneity for C-peptide^9^. The adipokine adiponectin, which is involved in energy balance and inflammation and lowered in obesity^10^, was inversely associated with CRC risk in a recent meta-analysis, but with large heterogeneity between studies^11^. Results for the adipokine leptin, an appetite regulator that is increased in obesity^10^, have been inconsistent^12^. The inconsistencies between studies of these metabolic biomarkers and CRC risk may be explained by differences in sample size, study design, study population (e.g., differences in geographic region, sex, age, or risk factor distributions), or biomarker type (e.g., total adiponectin vs high molecular weight adiponectin). Yet, such factors only partially explain the observed heterogeneity in meta-analyses^9,11,12^.

Heterogeneity in colorectal carcinogenesis could potentially contribute to the inconsistent associations reported between biomarkers of insulin resistance and adipokines and CRC risk. CRC develops through distinct pathways resulting in tumor subtypes with substantial differences in molecular and clinical characteristics^13,14^, and potentially also risk factors^15^. Studies of the association between BMI and molecular subtypes of CRC defined by key molecular tumor features such as *KRAS* and *BRAF* mutation status or MSI status have been inconclusive^16–22^. In the one previous biomarker study on the topic, a nested case-control study of 307 CRC cases, an inverse association was observed between plasma adiponectin and *KRAS*-mutated CRC risk^23^. If this subtype-specific association is confirmed, it would support differences in *KRAS*-mutation frequencies between study populations as a putative contributor to the inconsistent adiponectin-CRC associations reported. Whether associations between leptin or insulin resistance biomarkers and CRC risk differ by molecular subtypes has, to our knowledge, not been investigated.

The relationship between insulin resistance and circulating adipokine levels and CRC risk is also complicated by the influence of the carcinogenic process on metabolism. Both insulin resistance and alterations in circulating adipokine levels can be induced by established tumors, for example as a consequence of tumor byproducts or inflammation^24^. Since CRC develops slowly^25,26^, even studies with a relatively long follow-up time between sampling and diagnosis may be affected by reverse causality, which could distort relationships or create spurious associations. Changes in circulating biomarkers during the time period leading up to CRC diagnosis, and the potential influence of undiagnosed tumors on biomarker levels, can be studied using repeated prediagnostic blood samples from future CRC cases and cancer-free controls. However, no such longitudinal analyses have been reported for biomarkers of insulin resistance or adipokines in CRC, probably reflecting a paucity of suitable study cohorts.

In this nested case-control study, we took advantage of the unique sample collections in the North Sweden Health and Disease Study (NSHDS), including repeated blood samples from a subset of participants taken approximately 10 years apart and up to 27 years before CRC case diagnosis. The cohort is population based and well representative of a northern European population. Using 1010 matched case-control pairs within the NSHDS, we investigated circulating levels of insulin, C-peptide, adiponectin, and leptin in relation to the risk of CRC, and molecular subtypes of CRC defined by *KRAS* and *BRAF* mutation status and MSI status in the tumor. The availability of repeated samples additionally allowed us to examine time trends in the biomarkers over the long prediagnostic phase.

## Results

### Baseline characteristics

The median age at baseline of the 1010 CRC cases and 1010 matched controls from the Northern Sweden Health and Disease Study (NSHDS) was approximately 56 years, and 48% were women (Table 1). There was a slightly lower proportion of never smokers in prospective CRC cases compared to controls (39% vs 45%, P = 0.04). Occupational and recreational physical activity and alcohol intake were similar between the groups. Cases had higher baseline BMI (P = 0.009) and blood pressure (P = 0.02 and 0.03, for systolic and diastolic blood pressure respectively). Cases had slightly higher C-peptide and lower adiponectin at baseline (P = 0.07 and 0.03, respectively), and no material difference in insulin and leptin (P = 0.25 and 0.43), compared to controls.

**Table 1:** Baseline characteristics of the study participants. Median (quartiles) or n (%).

Variable	Controls (n = 1010)	CRC cases (n = 1010)	P^a^	Missing, n (%)
**Time from sampling to diagnosis, years**	—	12.3 (7.5–16.6)		0 (0)
**Age, years**	56.3 (49.9–60.0)	55.9 (49.9–60.0)		0 (0)
**Age groups, years**				0 (0)
30–39	23 (2)	23 (2)		
40–49	124 (12)	123 (12)		
50–59	367 (36)	370 (37)		
≥60	496 (49)	494 (49)		
**Sex, women**	485 (48)	485 (48)		0 (0)
**Smoking status**			0.04	43 (2)
Non-smoker	443 (45)	389 (39)		
Ex-smoker	321 (33)	359 (36)		
Current smoker	223 (23)	242 (24)		
**Occupational physical activity**			0.08	348 (17)
1 (sedentary or standing work)	213 (25)	202 (24)		
2 (light but partly physically active)	140 (17)	178 (21)		
3 (light and physically active)	217 (26)	184 (22)		
4 (sometimes physically strenuous)	222 (27)	218 (26)		
5 (physically strenuous most of the time)	45 (5)	53 (6)		
**Recreational physical activity**			0.50	41 (2)
1 (never)	379 (39)	420 (42)		
2 (every now and then – not regularly)	263 (27)	246 (25)		
3 (1–2 times/week)	187 (19)	197 (20)		
4 (2–3 times/week)	98 (10)	88 (9)		
5 (>3 times/week)	52 (5)	49 (5)		
**Alcohol intake, grams/day**	2.4 (0.3–6.0)	2.3 (0.2–5.9)	0.67	298 (15)
**Alcohol intake groups**			0.76	298 (15)
Zero intake	73 (8)	69 (8)		
Below median intake	398 (46)	414 (48)		
Above median intake	389 (45)	379 (44)		
**BMI, kg/m**^**2**^	25.5 (23.2–28.1)	26.0 (23.7–28.4)	0.009	20 (1)
**Systolic blood pressure, mmHg**	130.0 (120.0–145.8)	132.0 (120.0–150.0)	0.02	30 (1)
**Diastolic blood pressure, mmHg**	80.0 (75.0–90.0)	84.0 (75.0–90.0)	0.03	32 (2)
**Fasting status, hours**				
≤4	35 (3)	35 (3)		
5–6	168 (17)	168 (17)		
7–8	9 (1)	9 (1)		
>8	798 (79)	798 (79)		
**Glucose, mmol/l**	5.3 (4.9–5.8)	5.4 (5.0–5.8)	0.11	118 (6)
**Glucose tolerence**^**b**^**, mmol/l**	6.6 (5.7–7.4)	6.7 (5.7–7.7)	0.34	211 (10)
**Total cholesterol, mmol/l**	6.0 (5.2–6.9)	6.0 (5.3–6.9)	0.11	31 (2)
**Triglycerides, mmol/l**	1.2 (0.9–1.8)	1.4 (1.0–1.9)	0.10	380 (19)
**Insulin resistance biomarkers**				
Insulin, ng/mL	0.23 (0.15–0.36)	0.24 (0.16–0.38)	0.25	0 (0)
C-peptide, ng/mL	1.23 (0.95–1.68)	1.29 (0.98–1.82)	0.07	0 (0)
**Adipose tissue derived-biomarkers**				
Adiponectin, mg/L	20.5 (13.9–30.4)	19.5 (13.4–28.7)	0.03	0 (0)
Leptin, ng/mL	3.26 (1.40–6.98)	3.19 (1.46–7.78)	0.43	0 (0)

^a^Paired Wilcoxon signed-rank test within the matched case sets for continuous variables, chi-square tests for categorical variables.

^b^Blood glucose level measured as part of a standardized oral glucose tolerance test, 2 hours after glucose load.

**Figure 1:**
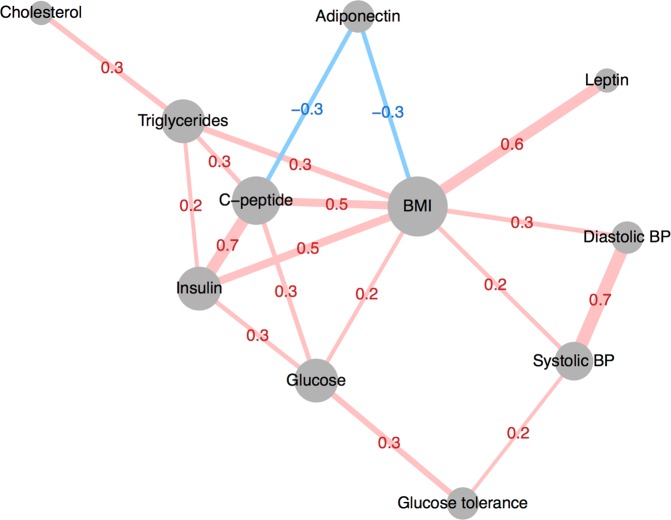
Correlation network of metabolic factors and biomarkers. Spearman’s correlation coefficients calculated in controls (n = 1010), adjusted for age, sex, and BMI. Absolute correlations above 0.2 are displayed. Node size corresponds to number of connections, edge thickness corresponds to the magnitude of the correlation. BP: Blood pressure.

Most metabolic factors and biomarkers were correlated with BMI (r = −0.3 to 0.6, Fig. 1). BMI-independent associations included correlations among insulin resistance-related factors (insulin, C-peptide, glucose, and glucose tolerance, r = 0.3 to 0.7), blood lipids (cholesterol and triglycerides, r = 0.3), and blood pressure (systolic and diastolic blood pressure, r = 0.7). Triglycerides were also correlated with insulin and C-peptide (r = 0.2 and 0.3), and adiponectin was correlated with C-peptide (r = −0.3).

### Baseline metabolic biomarker concentration and CRC risk

**Figure 2:**
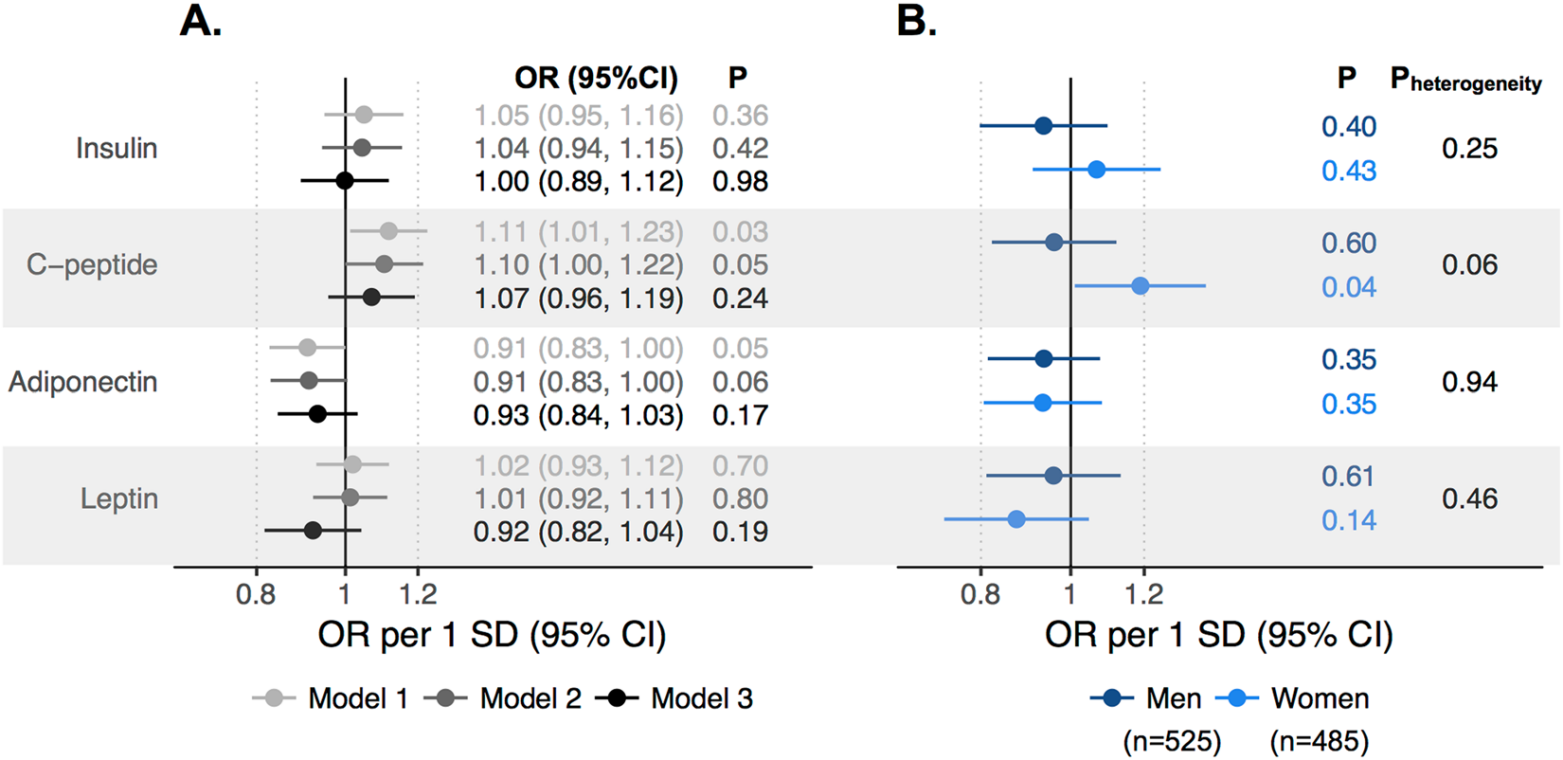
Odds ratios (ORs) for CRC risk per 1 SD increase in metabolic biomarkers in (**A**) all participants and (**B**) by sex. Estimates from conditional logistic regression models in 1010 cases and 1010 matched controls, adjusted only for matching variables age, sampling year, sex, cohort, and fasting status (Model 1), additionally adjusted for smoking, occupational and recreational physical activity, and alcohol intake (Model2), and additionally adjusted for BMI (Model 3). Sex-specific estimates were adjusted for the covariates included in Model 3. Heterogeneity by sex was tested with Wald’s test. Log-biomarker mean (SD), Insulin: −1.46 (0.81), C-peptide 0.27 (0.50), Adiponectin: 2.99 (0.57), Leptin: 1.18 (1.16).

Higher circulating levels of C-peptide and lower levels of adiponectin were weakly associated with an increased risk of CRC (ORs per 1 SD increase (95% CI): 1.11 (1.01, 1.23) and 0.91 (0.83, 1.00), respectively, Fig. 2A). Adjusting for smoking, physical activity, and alcohol intake variables had little effect on the risk estimates. Adjusting for BMI attenuated the risk estimates for both biomarkers (ORs per 1 SD increase (95% CI) in: C-peptide 1.07 (0.96, 1.19) and adiponectin 0.93 (0.84, 1.03)). Insulin and leptin were not associated with CRC risk in any model. There were no strong indications of non-linear associations for any biomarker (Supplementary Fig. 1). Associations for C-peptide were more prominent in women, even with BMI adjustment (OR_men_: 0.96 (0.82, 1.12) and OR_women_: 1.19 (1.01, 1.40), P_heterogeneity_ = 0.06, Fig. 2B). The ORs for insulin, adiponectin, and leptin and CRC risk were similar between men and women (P_heterogeneity_ > 0.20). There were no clear patterns in analyses stratified by follow-up time between blood sampling and diagnosis or by tumor site (all P_heterogeneity_ > 0.1, Supplementary Fig. 2).

### Longitudinal changes in metabolic biomarkers and CRC risk

Characteristics of the case participants with repeated measurements before diagnosis and matched controls (n = 518) are presented in Table 2. The median age for these participants was 50 at the first, baseline, measurement and 59.9 at the second, repeat, measurement taken closer to case diagnosis. The proportion of current smokers decreased significantly between the baseline and repeat measurements for both cases and controls. Both the cases and control groups demonstrated increases in BMI, as well as most other metabolic factors. There were no significant changes in the physical activity variables or alcohol intake (data not shown). Change in BMI over time was correlated with change in all biomarkers (insulin r = 0.3, C-peptide r = 0.4, adiponectin r = −0.3, and leptin r = 0.6). Intra-class correlation coefficients (ICC) of the biomarkers estimated in controls were for insulin: 0.49, C-peptide: 0.56, adiponectin: 0.83, and leptin 0.82.

**Table 2:** Baseline and follow-up characteristics of participants with repeated blood samples. Median (quartiles) or n (%).

Variable	Baseline (n = 518)	Repeat (n = 518)	P^a^
**CRC cases**	259 (50)	259 (50)	
**Time from sampling to diagnosis, years**	15.6 (12.9–19.0)	5.8 (2.9–9.1)	
**Age, years**	50.0 (40.5–50.2)	59.9 (51.0–60.1)	
**Sex, women**	226 (44)	226 (44)	
**Smoking status**			
Cases			0.0008
Non-smoker	97 (38)	112 (44)	
Ex-smoker	82 (32)	103 (41)	
Current smoker	74 (29)	39 (15)	
Controls			0.003
Non-smoker	110 (43)	114 (45)	
Ex-smoker	80 (32)	103 (41)	
Current smoker	63 (25)	34 (14)	
**BMI, kg/m2**			
Cases	25.3 (23.4–27.6)	26.4 (24.3–29.1)	<0.0001
Controls	24.6 (22.8–26.9)	26.0 (23.4–28.4)	<0.0001
**Systolic blood pressure, mmHg**			
Cases	126.5 (115.0–139.0)	134.0 (120.0–150.0)	<0.0001
Controls	122.0 (115.0–138.0)	135.0 (120.0–148.0)	<0.0001
**Diastolic blood pressure, mmHg**			
Cases	80.0 (72.0–88.0)	83.0 (75.0–93.0)	<0.0001
Controls	80.0 (71.2–86.0)	81.0 (75.0–90.0)	<0.0001
**Glucose, mmol/l**			
Cases	5.3 (5.0–5.7)	5.5 (5.2–6.0)	<0.0001
Controls	5.3 (4.9–5.7)	5.5 (5.1–6.0)	<0.0001
**Glucose tolerence**^**b**^**, mmol/l**			
Cases	6.3 (5.6–7.0)	6.9 (6.0–7.7)	<0.0001
Controls	6.2 (5.5–7.2)	6.7 (6.0–7.5)	<0.0001
**Total cholesterol, mmol/l**			
Cases	6.0 (5.1–6.6)	5.7 (5.0–6.3)	0.01
Controls	5.9 (5.2–6.8)	5.8 (5.1–6.5)	0.10
**Triglycerides, mmol/l**			
Cases	1.2 (0.9–1.8)	1.3 (0.9–1.7)	0.90
Controls	1.1 (0.8–1.7)	1.2 (0.9–1.7)	0.03

^a^Paired Wilcoxon signed-rank test within individuals for continuous variables, chi-square tests for categorical variables.

^b^Blood glucose level measured as part of a standardized oral glucose tolerance test, 2 hours after glucose load.

**Figure 3:**
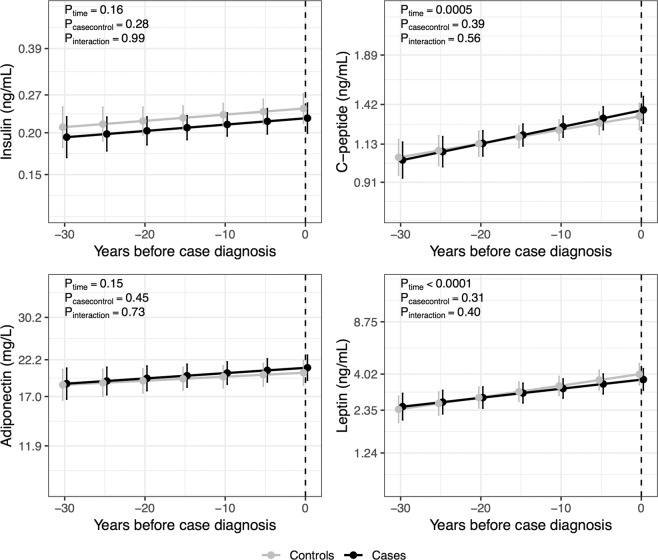
Within-individual changes in biomarker concentrations over time before diagnosis in CRC cases and matched controls. Marginal effects of time and 95% CIs estimated in 259 cases and 259 matched controls with matched repeated measurements before case diagnosis using linear mixed models, including participant ID and case set as random factors, and time until case diagnosis, case–control status, the interaction term between time and case–control status, smoking, occupational and recreational physical activity, alcohol intake, and BMI as fixed factors.

Within-individual biomarker changes in participants with repeated measurements sampled before case diagnosis, estimated using mixed models, are displayed in Fig. 3. C-peptide and leptin increased during prediagnostic time period (P_time_ = 0.0005 and P_time_ < 0.0001, respectively), with similar time trajectories in cases and matched controls (P_interaction_ = 0.56 and 0.40). Insulin and adiponectin did not change over time in cases or controls. Differences were similar regardless of stage at diagnosis (data not shown). The increase in biomarker concentrations over time occurred mainly in men, also with no difference by case-control status (data not shown).

### Metabolic biomarkers and molecular subtypes of CRC

Subtypes of CRC cases by *KRAS* and *BRAF* mutation status displayed expected clinical and molecular characteristics (Supplementary Table 1). *BRAF*-mutated cases were generally older at diagnosis, more often women, and more often had tumors situated in the right-sided colon and with MSI. *KRAS* and *BRAF* wild-type cases were slightly younger at diagnosis and more often had rectal tumors compared to the *KRAS*-mutated cases.

**Figure 4:**
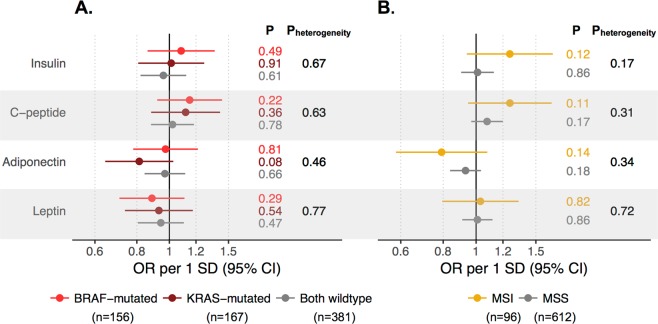
Odds ratios (ORs) of CRC by (**A**) *KRAS* and *BRAF* mutation status and (**B**) MSI status per 1 SD increase in metabolic biomarkers. Estimated in 1010 cases and 1010 matched controls, adjusted for matching variables and smoking, occupational and recreational physical activity, alcohol intake, and BMI. Heterogeneity across molecular subtypes was tested with a likelihood ratio test, comparing a model in which the risk association could vary across subtypes to a model were all associations were held constant. Numbers (n) within subtypes represent complete cases, molecular data for the remaining cases were imputed.

ORs for the risk of molecular subtypes of CRC defined by *KRAS* and *BRAF* mutation status and MSI status CRC, per SD increase in metabolic biomarkers, are presented in Fig. 4. Although the risk estimate for adiponectin was lower for *KRAS*-mutated CRC compared to the other subtypes (OR per 1 SD increase (95% CI): 0.81 (0.64, 1.03) for *KRAS*-mutated, 0.97 (0.78, 1.22) for *BRAF*-mutated, and 0.97 (0.84, 1.11) for double wild type, Fig. 4A), none differed significantly from 1, and there was no significant heterogeneity in risk estimates between subtypes (P_heterogeneity_ = 0.46). All other biomarkers had similar associations with CRC risk regardless of *KRAS*/*BRAF* subtype (P_heterogeneity_ = 0.63 to 0.77). No biomarker association differed by subtypes defined by MSI status (P_heterogeneity_ = 0.17 to 0.72, Fig. 4B). Sex-specific analyses displayed similar patterns (Supplementary Fig. 3). Estimates from complete-case analyses were similar to estimates from the multiple imputation estimates (Supplementary Table 2).

## Discussion

In this population-based, nested case-control study of 1010 CRC cases and 1:1 matched controls, higher C-peptide and lower adiponectin were weakly associated with an increased risk of developing CRC, whereas associations for insulin and leptin were null. In 518 participants with repeated measurements, biomarkers tended to increase over time during the prediagnostic period, but the changes were similar in cases and controls. Though not statistically significant, the results of the molecular subtype analyses were in line with a previous report suggesting an inverse association between adiponectin and the risk of *KRAS*-mutated CRC^23^.

The most recent meta-analyses of studies on circulating insulin, C-peptide, and adiponectin and CRC risk support associations, but with moderate to large heterogeneity between studies^9,11^. For leptin, meta-analyses report inconclusive results^12^. All previous investigations of these biomarkers and CRC risk have included less than 550 CRC cases, with the exception of two studies on adiponectin and leptin in the European Prospective Investigation into Cancer and Nutrition (EPIC) cohort, with approximately 1200 cases, reporting null associations after adjusting for BMI^27,28^. Given the heterogeneity among previous studies, the weak or null associations between biomarkers of insulin resistance and adipokines and CRC risk in our study are perhaps not surprising.

The potential association between C-peptide and CRC risk in women in our study was not observed in the most recent meta-analysis^9^. However, this may be due to the low combined sample size of women compared to men in the sex-specific analysis in that analysis (459 female CRC cases, 1233 male CRC cases)^9^, compared to the 485 female CRC cases in the present analysis. C-peptide is produced by beta cells in equal amounts as insulin^29^. The somewhat differing risk relationships for circulating insulin and C-peptide concentrations in our study may, therefore, seem contradictory. However, circulating C-peptide has a relatively long half-life^30^, and is a better marker for long-term insulin production than circulating fasting insulin.

Lower plasma adiponectin concentrations were associated with an increased risk of *KRAS*-mutated, and not *KRAS* wild type, CRC in a previous investigation of 307 CRC cases and 2:1 matched controls nested within the Nurses’ Health Study and Health Professionals Follow-up Study^23^. In the current study, the most distinct subtype-specific association observed was also an inverse relationship between adiponectin and *KRAS*-mutated CRC, though of smaller magnitude and not statistically significant. Differences in sample size or follow-up time (median 12.3 years vs. 8 years in the American study) between the studies might contribute to the weaker findings in the present investigation. The *KRAS*-mutation frequency was lower in our study (24% vs. 44% in the American study), though the absolute numbers of *KRAS*-mutated cases were comparable (n = 167 *KRAS*-mutated cases vs. n = 136 in the American study). Reported baseline BMI was similar, albeit slightly higher, in our study (mean BMI in controls 26.4/25.6 kg/m^2^ in men/women versus 25.3/24.7 kg/m^2^ in the American study). Thus, although our findings add some support for an influence of adiponectin in *KRAS*-driven colorectal carcinogenesis, additional confirmatory findings are required.

Furthermore, in this study, as in most previous studies, we assessed total adiponectin and not high molecular weight (HMW) and non-HMW separately. HMW and non-HMW have different biochemical characteristics, such that the HMW form is associated with insulin resistance, while non-HMW forms are inversely associated with inflammation^31,32^. Aleksandrova *et al*. studied the association of total, HMW, and non-HMW adiponectin in relation to CRC risk in the large EPIC cohort and found that only the non-HMW form was significantly associated with CRC risk after adjusting for potential mediators such as BMI and waist-to-hip-ratio, while total adiponectin was weakly associated to CRC risk before, but not after, adjustments^27^. These previous results for total adiponectin are similar to ours, and taken together they do not rule out a role for non-HMW adiponectin as a risk factor for CRC^11^, or an association to distinct molecular subtypes.

To our knowledge, this was the first study of insulin-resistance markers or leptin in relation to the risk of molecular subtypes of CRC. We found no statistically significant associations between insulin, C-peptide or leptin and any clinical or molecular subtype. Subgroup sample size may have limited the detection of small to moderate risk associations but, taken together, our findings do not support a major role for these biomarkers in the development of specific subtypes of CRC based on *KRAS* and *BRAF* mutations or MSI status in the tumor. As such, differences in molecular tumor traits between study populations probably cannot explain the previously reported varying results for insulin-resistance biomarkers and leptin in relation to CRC risk.

Previous longitudinal or retrospective observational studies have observed associations between adult weight gain and an increased risk of CRC^33^. Moreover, a longer duration of adulthood overweight and obesity is associated with a higher risk of CRC^34^. In our study, longitudinal analyses yielded results similar to those using only baseline measurements. Temporal changes in BMI, over a 10-year interval, were correlated with changes in metabolic biomarkers, and both changes in BMI and biomarker concentrations over time were similar in cancer cases and controls over a long prediagnostic period (median 15.6 years from baseline to diagnosis in cases with repeated measures). Furthermore, risk analyses stratified by follow-up time from sampling to diagnosis showed no signs of heterogeneity for any of the biomarkers. Taking together, our results suggest no large influence of undiagnosed CRC on circulating concentrations of these biomarkers, and do not support increasing CRC risk with a steeper increase in metabolic dysfunction over time.

The main limitation of this study was the lack of molecular tumor data for a portion of the CRC cases (approximately 30%), which could lead to selection bias. Tumor data availability depended on observable characteristics such as tumor site and stage. Therefore, we used observed data to impute plausible data using multiple imputation through chained equations, which can produce unbiased results in cases of missing at random data (i.e., when all predictors of missing status are measured and included in the imputation model)^35^. We cannot be sure that all predictors of missing status were accounted for, to meet the missing-at-random assumption, but results were very similar in analyses using imputed and complete-case data. There are also other ways to subtype CRC than by selected molecular features as in our study, such as using transcriptomics^13^, other aspects of the somatic mutational profile^36,37^, and features of the tumor microenvironment including, for example, the immune profile^38^. Also, histopathological data such as tumor grade and growth pattern were not available in our data set. More in-depth tumor phenotyping would, therefore, allow for a more comprehensive evaluation of subtype-specific relationships between metabolic biomarkers and CRC risk. However, the subtypes analyzed in our study are clinically relevant, represent distinct, non-overlapping subgroups, and have been observed to differ in risk factor associations^15,39^. Finally, despite the relatively large sample size in the study, the requirement of both prediagnostic exposure data and tumor data from the same patients limited the numbers in the smallest molecular subgroups, reducing statistical power. Consequently, although no subtype-specific associations between metabolic biomarkers and CRC risk were statistically significant, minor differences between subtypes may still exist.

One of the main strengths of our investigation was the study design, using prediagnostic exposure data and blood biomarker concentrations from a large set of CRC cases and matched controls including repeated measurements, collected on average 10 years apart. This allowed us to assess the temporal stability of biomarker status, as well as to investigate prediagnostic time trajectories in cancer cases compared to matched controls without cancer. All blood samples were collected and handled according to strict, high-quality protocols, including more than 8 hours of fasting for 79% of the participants in this study. Fasting status was also a matching criterion for case-control pairs. All biomarker analyses yielded low CVs. The availability of prediagnostic blood samples and molecular tumor data from the same CRC patients allowed us to investigate the association between metabolic mediators in relation to molecular tumor subtypes. Since pathogenic mechanisms are more homogenous within such CRC subtypes compared to CRC as a whole, results from molecular pathological studies such as ours can provide new etiological insights, which might be masked when CRC is investigated as a single disease. The recruitment base for the VIP, making up the majority of the NSHDS participants, is the entire population of the Swedish county of Västerbotten, with a participation rate of around 70% and no indication of a large selection^40^. Although rates of overweight and obesity are lower in Sweden than in the United States (Gomez 2017), a quarter of the participants had a BMI over 28, providing a sufficient range of body sizes for the analyses. Follow-up through the essentially complete Swedish cancer registry^41^ demonstrates nearly identical CRC incidence rates in the cohort and the underlying population. Thus, the population-based nature of the NSHDS reduced the risk of selection bias in the study. Finally, the long median follow-up time between blood sampling and diagnosis, median 12.3 years, allowed for time-stratified analyses, reducing the risk of reverse causation.

To conclude, this study, the first to assess multiple, prediagnostic, metabolic biomarkers in relation to the risk of molecular CRC subtypes, nuances the current understanding of the role of body size and metabolism in colorectal carcinogenesis. We observed an inverse association between adiponectin and the risk of *KRAS*-mutated CRC which, although not statistically significant, was consistent with the one previous report on the topic. But in general, circulating biomarkers of insulin resistance and adipokines were at most weakly associated with CRC risk, and not clearly associated with specific subtypes of CRC based on *KRAS*- and *BRAF*-mutations and MSI status of the tumor.

## Methods

### Study population

This was a case-control study nested within two prospective population-based cohorts of the Northern Sweden Health and Disease Study (NSHDS): the Västerbotten Intervention Programme (VIP) and the MONICA project. Both cohorts have been described in detail elsewhere^40,42^. In short, the VIP is an ongoing health screening intervention in which all residents of the Västerbotten County in Sweden are invited to general health exams at 10-year intervals starting at 40 years^40^. They also donate a blood sample for biobank research and fill out an extensive questionnaire on health and lifestyle. The average participation rate is 66%, with no indication of a major selection^40^. The MONICA consists of randomly selected 25 to 74-year-olds living in northern Sweden (Västerbotten and Norrbotten County) invited to participate in six health surveys between 1986–2014 (average participation rate 74%)^42^. At the final date of entrance to this study (January 19, 2016), the NSHDS included 119 738 participants with 183 699 observations.

### Ethical considerations

The study protocol was approved by the Research Ethics Committee of Umeå University, Umeå, Sweden. All participants gave a written informed consent. All analyses were conducted in accordance with relevant guidelines and regulations.

### Study participants

Prospective CRC cases up until May 31, 2016, were identified by linkage to the near-complete Swedish national registries, for whatever occurred first of cancer diagnosis other than non-melanoma skin cancer (Cancer Registry of Northern Sweden), death (Swedish Cause of Death Registry), or emigration (Swedish Registry of Total Population and Population Changes). Participants diagnosed with colorectal adenocarcinoma were identified using ICD-10 C18.0 and C18.2–18.9 for colon, C19.9 and C20.9 for rectal cancers. CRC diagnoses were verified, and data on tumor stage and anatomical site were collected, by linkage to the national Swedish Colorectal Cancer Registry or from medical records by a gastrointestinal pathologist. A total of 1013 cases with an available prediagnostic blood sample was identified. After excluding cases with nonmatching blood sampling and questionnaire dates (n = 3), 1010 cases remained. The median time between baseline blood sampling and diagnosis of the cases was 12.3 years. A total of 8 cases had unknown tumor site (1%), and 64 had unknown tumor stage (6%). For each of the 1010 cases, one control matched by cohort, sex, age at and year of blood sampling and data collection (±1 year), and fasting status was randomly selected. All controls had to be alive and with no diagnosed cancer other than non-melanoma skin cancer at the time of diagnosis of their corresponding case.

Of the 1010 cases, 265 had repeated prediagnostic blood samples. For these cases, the controls were also selected on a 1:1 basis, i.e. requiring matched repeated samples from the controls, and using the same matching criteria as for the cases with single samples. Measurements taken less than 5 years apart were excluded (n = 6), leaving 259 cases and 259 controls for data analysis (median time between measurements: 9.9 years, quartiles: 9.7 to 10.1 years). For the repeated samples, the median time between sampling and case diagnosis was 15.6 years for the baseline measurement, and 5.8 years for the repeat measurement.

### Health examination

Very similar protocols for the health examination were used in the VIP and MONICA cohorts. Participants’ height and weight were measured in light clothing without shoes. Systolic and diastolic blood pressure were measured once with a mercury sphygmomanometer after a 5-minute rest in the supine position until 2009, and from 2009 in the sitting position. Blood pressure measurements before and after 2009 in the VIP were normalized using formulas provided by the cohort. Questionnaire items used in this study included information on smoking (self-reported non-smoker, ex-smoker, or current smoker), occupational physical activity (self-reported on a scale from 1–5, from sedentary to physically active occupation), recreational physical activity (self-reported on a scale from 1–5, from no physical activity to >3 times a week), and alcohol intake (self-reported from a validated food frequency questionnaire^43^: zero intake, above/below sex and questionnaire-version-specific median of self-reported intake in grams/day).

### Blood sampling and biomarker analyses

In the VIP, venous blood samples are collected in the morning following an overnight fast, but non-complying participants may also provide a sample. In the MONICA, venous blood samples were collected after fasting for a minimum of 4 hours up until 1992, and 8 hours after 1992. In this study, 79% of the participants had fasted for more than 8 hours and only 3% less than 4 hours. Blood samples in both cohorts were collected in EDTA and heparin tubes using a standardized protocol, separated into plasma, buffy coat, and erythrocyte fractions, aliquoted and cryopreserved at −80 °C within 1 hour of collection, or at −20 °C for at most 1 week before storage at −80 °C.

Glucose concentrations were analyzed in plasma with and without a 2-hour 75 g oral glucose load (to test glucose tolerance) with a Reflotron bench-top analyzer (Roche Diagnostics) until 2004, and from 2004 with a Hemocue bench-top analyzer (Quest Diagnostics). Total cholesterol and triglyceride concentrations were analyzed with a Reflotron bench-top analyzer until 2009, and from 2009 with an enzymatic method at the clinical chemistry laboratory at the nearest hospital. All MONICA samples were analyzed using the methods used after 2009. Blood lipid measurements before and after 2009 in the VIP were normalized using formulas provided by the cohort.

Concentrations of insulin, C-peptide and leptin in EDTA plasma were measured in pre-coated 96-well plates using a custom-designed multiplex immunoassay (7-Spot Prototype Human Metabolic 5-plex) from Meso Scale Discovery (MSD Rockville MD). Adiponectin in plasma was measured separately using the same system (Human Adiponectin Kit, MSD Rockville MD). All assays were run according to the manufacturer’s instructions with all reagents from MSD. Briefly, prepared Blocker A solution was added to the plates, which were placed on a shaker at room temperature for one hour. After this, 25 μl undiluted sample +25 μl working solution was added to the multiplex plates and 10 μl diluted sample (1/1000) +40 μl Diluent 12 to the adiponectin plates. Standards and pooled plasma controls were added in duplicate to all plates. Plates were incubated on a shaker at room temperature for two hours. Detection antibody solution was added to the plates, which were incubated for an additional hour on a shaker after which Reading buffer was added, and plates were immediately read on a MESO QuickPlex SQ 120 (MSD Rockville MD). Plates were washed 3 times between all steps. Matched case sets were analyzed together, in random order, on the same analysis plate. Investigators and laboratory staff were blinded to case and control status until the data preprocessing and analysis phase. Inter- and intra-assay coefficients of variation (CVs) calculated on pooled plasma control samples were low for all biomarkers (inter/intra-CV (%): insulin (3.2/0.7), C-peptide (2.7/1.4), adiponectin (1.4/0.5), leptin (1.5/0.4). There was an indication of laboratory drift for insulin and leptin. Therefore, these biomarkers were normalized to the last plate (plate 30) by multiplying measurements with the control sample ratio according to the formula: $Y{{\prime} }_{i,b}={Y}_{i,b}\,\ast \,\,\frac{{\bar{C}}_{b}}{{{\bar{C}}_{30}}^{{\prime} }}$, where *Y*′_*i,b*_ and *Y*_*i,b*_ are the adjusted and unadjusted measurements, respectively, for individual *i* in batch *b*, and ${\bar{C}}_{b}$ and ${\bar{C}}_{30}$ are the mean of the control sample measurements in batch *b* and 30, respectively. Results were also calculated using plate-specific standardized variables, with similar results. Therefore, the normalized data were used to generate the study results. Biomarker measurements below the curve range (0 to 2% of samples per biomarker) were assumed to be low and were therefore replaced with the plate-specific minimum concentration.

### Tumor tissue analyses

DNA was extracted and purified using a Qiagen QIAamp DNA FFPE Tissue Kit from formalin-fixed, paraffin-embedded tumor tissue collected during routine clinical practice at the Department of Clinical Pathology, Umeå University Hospital, Umeå, Sweden. In total, 841 cases (83%) had available tumor tissue. *KRAS* was analyzed by sequencing the activating mutations in codon 12 and 13 using Big Dye v. 3.1, according to the manufacture protocol (Applied Biosystems, Life Technologies, Foster City, CA, USA)^44^. The mutational status of *BRAF*^V600E^ was analyzed using the TaqMan allelic discrimination assay (reagents from Applied Biosystems) or, later, with digital droplet PCR (reagents from Bio-Rad Laboratories, Hercules, CA, USA)^45^. As *KRAS* and *BRAF* are generally considered mutually exclusive within a clone, CRC cases were classified as *KRAS*-mutated, *BRAF*-mutated, or *KRAS*/*BRAF* wild type. Cases with mutations in both *KRAS* and *BRAF* (n = 5, 0.6% of the cases with available tumor tissue) were excluded in the subtype analyses. For cases diagnosed up to 2009, microsatellite instability (MSI) status was determined using immunohistochemical analysis of MLH1, MSH2, MSH6, and PMS2. Samples lacking tumor cells with nuclear staining for ≥1 of the mismatch repair proteins were categorized as MSI^46^. From 2009, a PCR-based method was used (Promega MSI Analysis System, Version 1.2, Madison, WI). A subset of cases (n = 70) was analyzed using both methods, with 100% concordance. Of the cases with tumor tissue available, 132 had inconclusive *KRAS* or *BRAF* mutation status, and 133 cases had inconclusive MSI status, mostly caused by lack of tumor DNA. In total, 387 cases had unavailable *KRAS*, *BRAF*, or MSI status data (n = 216 had unavailable data on all molecular features). The cases with unavailable data were more often diagnosed at younger ages and with distal tumors compared to cases with available data (Supplementary Table 3).

### Statistical analyses

Missing baseline, repeated measure and tumor data were assumed to be missing at random and therefore imputed using multiple imputation by chained equations with the mice R-package^35^. Twenty data sets were imputed, in 25 iterations, with a predictive mean matching model for continuous variables and logistic regression models for categorical variables. Models included data on all biomarkers and covariates as well as age, sex, cohort (VIP or MONICA), fasting status (≤4, 5–6, 7–8, >8 hours), smoking, occupational and recreational physical activity, alcohol intake, and BMI as predictors. For each of the 20 imputed data sets, missing data on tumor stage, tumor site, *KRAS* and *BRAF* mutation status, and MSI status in cases were imputed in 40 imputed data sets in 25 iterations including the same predictors as before, as well as the tumor variables and age at and year of diagnosis (1986–2006, 2007–2012, 2013–2016). We graphically checked for convergence of all imputed values across iterations. We also ensured the plausibility of the imputed variables by comparing distributions between imputed and non-missing values. All statistical analyses were run separately on the imputed datasets and then aggregated using Rubin’s rules^47^.

Partial correlations between metabolic factors and biomarkers adjusted for age, sex, and BMI were calculated with Spearman’s correlations coefficient on residuals from regression models including the adjustment variables for each pair of biomarkers.

Associations between biomarkers and CRC risk were evaluated by estimating odds ratios (ORs) per 1 standard deviation (SD) increase in biomarkers concentrations by modeling standardized biomarkers (within sex) in conditional logistic regression models conditioned on the matched case sets. Covariates to include in the multivariable models were chosen based on previous evidence for a relationship between the covariate and biomarker and CRC risk. For each biomarker, three models were fitted: Model 1 included only the matching variables, Model 2 additionally included smoking, occupational and recreational physical activity, and alcohol intake, and Model 3 additionally included BMI. To check for nonlinear associations, continuous variables were modeled using restricted cubic splines (with knots at the 5^th^, 50^th^, 95^th^ percentiles). Nonlinearity was tested with a likelihood ratio test comparing the spline model to a linear model. To evaluate whether associations between exposures and CRC risk differ by follow-up time between blood sampling and diagnosis, anatomical subsite, or molecular subtypes, we estimated subtype-specific ORs with conditional logistic regression models with a competing risks approach using the duplication method^48^. Heterogeneity was tested with a likelihood ratio test, comparing a model in which the risk association could vary across subtypes to a model in which all associations were held constant.

For participants with repeated measurements, we modeled changes in biomarkers over time in cases and controls using linear mixed models. Log-transformed biomarker concentrations were modeled including participant ID and case set as random factors, and case-control status, time until case diagnosis (time = 0 at diagnosis), smoking, occupational and recreational physical activity, alcohol intake, and BMI as fixed factors. To test for differences in biomarker changes over time between cases and control, we included an interaction term between case-control status and time. Models were fitted using the lme4 R-package. Associations were tested using regression coefficient t-tests with degrees of freedom from Satterthwaite’s approximation.

All computations were conducted in R v.3.5.0 (R Foundation for Statistical Computing, Vienna, Austria). All tests were 2-sided when applicable. We applied a conservative significance threshold of 0.005 for analyses which were not primarily descriptive or confirmatory^49^.

## Supplementary information

Supplementary material.

## References

[1] Bhaskaran K (2014). Body-mass index and risk of 22 specific cancers: a population-based cohort study of 5.24 million UK adults. Lancet.

[2] Bardou M, Barkun AN, Martel M (2013). Obesity and colorectal cancer. Gut.

[3] Jarvis D (2016). Mendelian randomisation analysis strongly implicates adiposity with risk of developing colorectal cancer. British journal of cancer.

[4] Gao C (2016). Mendelian randomization study of adiposity-related traits and risk of breast, ovarian, prostate, lung and colorectal cancer. Int J Epidemiol.

[5] Aleman JO (2014). Mechanisms of obesity-induced gastrointestinal neoplasia. Gastroenterology.

[6] Giouleme O, Diamantidis MD, Katsaros MG (2011). Is diabetes a causal agent for colorectal cancer? Pathophysiological and molecular mechanisms. World journal of gastroenterology: WJG.

[7] Moon HS (2013). Salutary effects of adiponectin on colon cancer: *in vivo* and *in vitro* studies in mice. Gut.

[8] Howard JM, Pidgeon GP, Reynolds JV (2010). Leptin and gastro-intestinal malignancies. Obesity reviews: an official journal of the International Association for the Study of Obesity.

[9] Xu Jinming, Ye Yao, Wu Han, Duerksen-Hughes Penelope, Zhang Honghe, Li Peiwei, Huang Jian, Yang Jun, Wu Yihua, Xia Dajing (2016). Association between markers of glucose metabolism and risk of colorectal cancer. BMJ Open.

[10] Meier U, Gressner AM (2004). Endocrine regulation of energy metabolism: review of pathobiochemical and clinical chemical aspects of leptin, ghrelin, adiponectin, and resistin. Clin Chem.

[11] Lu W, Huang Z, Li N, Liu H (2018). Low circulating total adiponectin, especially its non-high-molecular weight fraction, represents a promising risk factor for colorectal cancer: a meta-analysis. OncoTargets and therapy.

[12] Gialamas SP (2013). Circulating leptin levels and risk of colorectal cancer and adenoma: a case-control study and meta-analysis. Cancer causes & control: CCC.

[13] Guinney J (2015). The consensus molecular subtypes of colorectal cancer. Nature medicine.

[14] Yamauchi M (2012). Colorectal cancer: a tale of two sides or a continuum?. Gut.

[15] Leggett B (2010). & Whitehall, V. Role of the serrated pathway in colorectal cancer pathogenesis. Gastroenterology.

[16] Slattery ML (2000). Associations between dietary intake and Ki-ras mutations in colon tumors: A population-based study. Cancer research.

[17] Slattery ML (2001). Lifestyle factors and Ki-ras mutations in colon cancer tumors. Mutation research.

[18] Slattery ML (2010). Diet, physical activity, and body size associations with rectal tumor mutations and epigenetic changes. Cancer causes & control: CCC.

[19] Brandstedt J (2014). Associations of anthropometric factors with KRAS and BRAF mutation status of primary colorectal cancer in men and women: a cohort study. PloS one.

[20] Hughes LAE (2012). Body size and risk for colorectal cancers showing BRAF mutations or microsatellite instability: a pooled analysis. Int J Epidemiol.

[21] Carr PR (2018). Lifestyle factors and risk of sporadic colorectal cancer by microsatellite instability status: a systematic review and meta-analyses. Annals of oncology: official journal of the European Society for Medical Oncology / ESMO.

[22] Myte Robin, Gylling Björn, Häggström Jenny, Häggström Christel, Zingmark Carl, Löfgren Burström Anna, Palmqvist Richard, Van Guelpen Bethany (2019). Metabolic factors and the risk of colorectal cancer by KRAS and BRAF mutation status. International Journal of Cancer.

[23] Inamura Kentaro, Song Mingyang, Jung Seungyoun, Nishihara Reiko, Yamauchi Mai, Lochhead Paul, Qian Zhi Rong, Kim Sun A, Mima Kosuke, Sukawa Yasutaka, Masuda Atsuhiro, Imamura Yu, Zhang Xuehong, Pollak Michael N., Mantzoros Christos S., Harris Curtis C., Giovannucci Edward, Fuchs Charles S., Cho Eunyoung, Chan Andrew T., Wu Kana, Ogino Shuji (2015). Prediagnosis Plasma Adiponectin in Relation to Colorectal Cancer Risk According toKRASMutation Status. Journal of the National Cancer Institute.

[24] Dev R, Bruera E, Dalal S (2018). Insulin resistance and body composition in cancer patients. Annals of oncology: official journal of the European Society for Medical Oncology/ESMO.

[25] Brenner H, Altenhofen L, Katalinic A, Lansdorp-Vogelaar I, Hoffmeister M (2011). Sojourn time of preclinical colorectal cancer by sex and age: estimates from the German national screening colonoscopy database. American journal of epidemiology.

[26] Brenner H, Altenhofen L, Stock C, Hoffmeister M (2013). Natural history of colorectal adenomas: birth cohort analysis among 3.6 million participants of screening colonoscopy. Cancer epidemiology, biomarkers & prevention: a publication of the American Association for Cancer Research, cosponsored by the American Society of Preventive Oncology.

[27] Aleksandrova K (2012). Total and high-molecular weight adiponectin and risk of colorectal cancer: the European Prospective Investigation into Cancer and Nutrition Study. Carcinogenesis.

[28] Aleksandrova K (2012). Leptin and soluble leptin receptor in risk of colorectal cancer in the European Prospective Investigation into Cancer and Nutrition cohort. Cancer research.

[29] Jones AG, Hattersley AT (2013). The clinical utility of C-peptide measurement in the care of patients with diabetes. Diabetic medicine: a journal of the British Diabetic Association.

[30] Van Cauter E, Mestrez F, Sturis J, Polonsky KS (1992). Estimation of insulin secretion rates from C-peptide levels. Comparison of individual and standard kinetic parameters for C-peptide clearance. Diabetes.

[31] Schraw T, Wang ZV, Halberg N, Hawkins M, Scherer PE (2008). Plasma adiponectin complexes have distinct biochemical characteristics. Endocrinology.

[32] Neumeier M (2006). Different effects of adiponectin isoforms in human monocytic cells. Journal of leukocyte biology.

[33] Karahalios A, English DR, Simpson JA (2015). Weight change and risk of colorectal cancer: a systematic review and meta-analysis. American journal of epidemiology.

[34] Arnold M (2016). Duration of Adulthood Overweight, Obesity, and Cancer Risk in the Women's Health Initiative: A Longitudinal Study from the United States. PLoS medicine.

[35] van Buuren S, Groothuis-Oudshoorn K (2011). mice: Multivariate Imputation by Chained Equations in R. J Stat Softw.

[36] Kandoth C (2013). Mutational landscape and significance across 12 major cancer types. Nature.

[37] Lawrence MS (2014). Discovery and saturation analysis of cancer genes across 21 tumour types. Nature.

[38] Taube JM (2018). Implications of the tumor immune microenvironment for staging and therapeutics. Mod Pathol.

[39] Ogino S, Chan AT, Fuchs CS, Giovannucci E (2011). Molecular pathological epidemiology of colorectal neoplasia: an emerging transdisciplinary and interdisciplinary field. Gut.

[40] Norberg Margareta, Wall Stig, Boman Kurt, Weinehall Lars (2010). The Västerbotten Intervention Programme: background, design and implications. Global Health Action.

[41] Barlow L, Westergren K, Holmberg L, Talback M (2009). The completeness of the Swedish Cancer Register: a sample survey for year 1998. Acta Oncol.

[42] Benckert M, Lilja M, Soderberg S, Eliasson M (2015). Improved metabolic health among the obese in six population surveys 1986 to 2009: the Northern Sweden MONICA study. BMC Obes.

[43] Johansson I (2002). Validation and calibration of food-frequency questionnaire measurements in the Northern Sweden Health and Disease cohort. Public health nutrition.

[44] Eklof V (2013). The prognostic role of KRAS, BRAF, PIK3CA and PTEN in colorectal cancer. British journal of cancer.

[45] Benlloch S (2006). Detection of BRAF V600E mutation in colorectal cancer: comparison of automatic sequencing and real-time chemistry methodology. J Mol Diagn.

[46] Van Guelpen B (2010). One-carbon metabolism and CpG island methylator phenotype status in incident colorectal cancer: a nested case-referent study. Cancer causes & control: CCC.

[47] Marshall A, Altman DG, Holder RL, Royston P (2009). Combining estimates of interest in prognostic modelling studies after multiple imputation: current practice and guidelines. BMC Med Res Methodol.

[48] Wang M (2016). Statistical methods for studying disease subtype heterogeneity. Statistics in medicine.

[49] Benjamin DJ (2018). Redefine statistical significance. Nat Hum Behav.

