# Supplementary material for: A longitudinal study of prediagnostic metabolic biomarkers and the risk of molecular subtypes of colorectal cancer
Source: Sci Rep. 2020 Mar 24;10:5336. doi: 10.1038/s41598-020-62129-1 (PMC7093429; doi:10.1038/s41598-020-62129-1)
Supplement: Supplementary file 1 — Supplementary material. [file 41598_2020_62129_MOESM1_ESM.pdf]

A longitudinal study of prediagnostic metabolic biomarkers and the risk of molecular subtypes of colorectal cancer
Myte et al.

SUPPLEMENTARY MATERIAL

Supplementary Table 1 Clinical characteristics in CRC cases by KRAS/BRAF mutation status.

Variable	BRAF-mutated (n=156)	KRAS-mutated (n=167)	Both wild type (n=381)	Pa	Missing, n (%)
**Age at diagnosis, years**	70.1 (65.5-70.1)	67.1 (58.1-67.1)	66.0 (60.1-66.0)	<0.0001	0 (0)
**Sex, women**	99 (63)	88 (53)	164 (43)	<0.0001	0 (0)
**Tumor site, n (%)**				<0.0001	8 (1)
Right colon	113 (72)	57 (34)	79 (21)		
Left colon	25 (16)	48 (29)	127 (34)		
Rectum	18 (12)	61 (37)	173 (46)		
**Tumor stage, n (%)**				0.35	65 (6)
Stage I&II	73 (48)	88 (56)	194 (53)		
Stage III&IV	79 (52)	69 (44)	170 (47)		
**MSI status, n (%)**				<0.0001	302 (30)
MSI	68 (50)	3 (2)	18 (5)		
MSS	68 (50)	137 (98)	324 (95)		

MSS: Microsatellite stable.

a Linear regression-based test for age at diagnosis, chi-square tests for other variables.

Supplementary Table 2 Odds ratios (95% CIs) of colorectal cancer (CRC) and CRC subtypes by metabolic biomarkers estimated in complete-case (CC) and multiple imputation (MI) analyses

Exposure	Outcome	Subtype	n	n_CC	n_cases_CC	HR_CC (95% CI)[a]	$P_{heterogeneity}$_CC	HR_MI (95% CI)[a]	$P_{heterogeneity}$_MI
Insulin	CRC risk		2020	2020	1010	1.00 (0.89, 1.12)		1.00 (0.89, 1.12)	
C-peptide	CRC risk		2020	2020	1010	1.07 (0.96, 1.19)		1.07 (0.96, 1.19)	
Adiponectin	CRC risk		2020	2020	1010	0.93 (0.84, 1.03)		0.93 (0.84, 1.03)	
Leptin	CRC risk		2020	2020	1010	0.93 (0.82, 1.05)		0.92 (0.82, 1.04)	
Insulin	CRC risk by follow-up time	<9 years	2020	2020	337	1.00 (0.84, 1.20)	0.722	1.00 (0.84, 1.19)	0.726
Insulin	CRC risk by follow-up time	9 to <15 years	2020	2020	336	1.05 (0.88, 1.26)	0.722	1.05 (0.88, 1.26)	0.726
Insulin	CRC risk by follow-up time	15 to <27 years	2020	2020	337	0.95 (0.79, 1.14)	0.722	0.95 (0.79, 1.14)	0.726
C-peptide	CRC risk by follow-up time	<9 years	2020	2020	337	1.07 (0.88, 1.29)	0.609	1.06 (0.87, 1.28)	0.602
C-peptide	CRC risk by follow-up time	9 to <15 years	2020	2020	336	1.14 (0.95, 1.38)	0.609	1.15 (0.95, 1.38)	0.602
C-peptide	CRC risk by follow-up time	15 to <27 years	2020	2020	337	1.02 (0.87, 1.19)	0.609	1.02 (0.87, 1.19)	0.602
Adiponectin	CRC risk by follow-up time	<9 years	2020	2020	337	0.91 (0.77, 1.08)	0.959	0.92 (0.78, 1.08)	0.969
Adiponectin	CRC risk by follow-up time	9 to <15 years	2020	2020	336	0.94 (0.79, 1.11)	0.959	0.93 (0.79, 1.11)	0.969
Adiponectin	CRC risk by follow-up time	15 to <27 years	2020	2020	337	0.95 (0.80, 1.12)	0.959	0.95 (0.80, 1.12)	0.969
Leptin	CRC risk by follow-up time	<9 years	2020	2020	337	0.86 (0.72, 1.02)	0.325	0.85 (0.72, 1.01)	0.316
Leptin	CRC risk by follow-up time	9 to <15 years	2020	2020	336	1.02 (0.85, 1.22)	0.325	1.01 (0.84, 1.21)	0.316
Leptin	CRC risk by follow-up time	15 to <27 years	2020	2020	337	0.92 (0.77, 1.11)	0.325	0.92 (0.76, 1.11)	0.316
Insulin	CRC risk by tumor site	Rectum	2020	2004	318	0.95 (0.79, 1.14)	0.131	0.96 (0.80, 1.15)	0.136
Insulin	CRC risk by tumor site	Right colon	2020	2004	296	1.16 (0.96, 1.41)	0.131	1.16 (0.96, 1.41)	0.136

Insulin	CRC risk by tumor site	Left colon	2020	2004	388	0.91 (0.77, 1.09)	0.131	0.91 (0.77, 1.09)	0.136
C-peptide	CRC risk by tumor site	Rectum	2020	2004	318	1.09 (0.92, 1.29)	0.842	1.09 (0.92, 1.29)	0.855
C-peptide	CRC risk by tumor site	Right colon	2020	2004	296	1.03 (0.86, 1.22)	0.842	1.03 (0.86, 1.22)	0.855
C-peptide	CRC risk by tumor site	Left colon	2020	2004	388	1.09 (0.91, 1.31)	0.842	1.09 (0.91, 1.31)	0.855
Adiponectin	CRC risk by tumor site	Rectum	2020	2004	318	1.00 (0.84, 1.18)	0.571	1.00 (0.85, 1.18)	0.565
Adiponectin	CRC risk by tumor site	Right colon	2020	2004	296	0.88 (0.73, 1.06)	0.571	0.88 (0.73, 1.06)	0.565
Adiponectin	CRC risk by tumor site	Left colon	2020	2004	388	0.91 (0.77, 1.07)	0.571	0.91 (0.78, 1.07)	0.565
Leptin	CRC risk by tumor site	Rectum	2020	2004	318	0.94 (0.79, 1.13)	0.929	0.94 (0.78, 1.12)	0.906
Leptin	CRC risk by tumor site	Right colon	2020	2004	296	0.94 (0.78, 1.14)	0.929	0.94 (0.78, 1.13)	0.906
Leptin	CRC risk by tumor site	Left colon	2020	2004	388	0.91 (0.76, 1.08)	0.929	0.90 (0.76, 1.06)	0.906
Insulin	CRC risk by KRAS and BRAF mutation status	BRAF-mutated	2020	1408	156	1.07 (0.84, 1.36)	0.485	1.08 (0.86, 1.36)	0.665
Insulin	CRC risk by KRAS and BRAF mutation status	KRAS-mutated	2020	1408	167	0.95 (0.75, 1.20)	0.485	1.01 (0.81, 1.27)	0.665
Insulin	CRC risk by KRAS and BRAF mutation status	Both wild type	2020	1408	381	0.90 (0.75, 1.08)	0.485	0.96 (0.82, 1.12)	0.665
C-peptide	CRC risk by KRAS and BRAF mutation status	BRAF-mutated	2020	1408	156	1.14 (0.91, 1.44)	0.394	1.15 (0.92, 1.44)	0.628
C-peptide	CRC risk by KRAS and BRAF mutation status	KRAS-mutated	2020	1408	167	1.04 (0.81, 1.34)	0.394	1.12 (0.88, 1.42)	0.628
C-peptide	CRC risk by KRAS and BRAF mutation status	Both wild type	2020	1408	381	0.95 (0.81, 1.13)	0.394	1.02 (0.88, 1.18)	0.628
Adiponectin	CRC risk by KRAS and BRAF mutation status	BRAF-mutated	2020	1408	156	0.94 (0.74, 1.20)	0.410	0.97 (0.78, 1.22)	0.455
Adiponectin	CRC risk by KRAS and BRAF mutation status	KRAS-mutated	2020	1408	167	0.83 (0.65, 1.05)	0.410	0.81 (0.64, 1.03)	0.455
Adiponectin	CRC risk by KRAS and BRAF mutation status	Both wild type	2020	1408	381	1.00 (0.86, 1.18)	0.410	0.97 (0.84, 1.11)	0.455
Leptin	CRC risk by KRAS and BRAF mutation status	BRAF-mutated	2020	1408	156	0.94 (0.75, 1.19)	0.865	0.89 (0.71, 1.11)	0.767
Leptin	CRC risk by KRAS and BRAF mutation status	KRAS-mutated	2020	1408	167	0.99 (0.77, 1.25)	0.865	0.93 (0.74, 1.17)	0.767
Leptin	CRC risk by KRAS and BRAF mutation status	Both wild type	2020	1408	381	1.01 (0.85, 1.21)	0.865	0.94 (0.81, 1.10)	0.767
Insulin	CRC risk by MSI status	MSS	2020	1416	612	0.92 (0.79, 1.06)	0.143	0.96 (0.85, 1.09)	0.142

Insulin	CRC risk by MSI status	MSI	2020	1416	96	1.19 (0.86, 1.64)	0.143	1.22 (0.91, 1.63)	0.142
C-peptide	CRC risk by MSI status	MSS	2020	1416	612	1.04 (0.90, 1.19)	0.421	1.04 (0.92, 1.17)	0.279
C-peptide	CRC risk by MSI status	MSI	2020	1416	96	1.19 (0.87, 1.65)	0.421	1.23 (0.92, 1.62)	0.279
Adiponectin	CRC risk by MSI status	MSS	2020	1416	612	0.98 (0.86, 1.11)	0.414	0.95 (0.85, 1.06)	0.343
Adiponectin	CRC risk by MSI status	MSI	2020	1416	96	0.84 (0.59, 1.18)	0.414	0.81 (0.59, 1.10)	0.343
Leptin	CRC risk by MSI status	MSS	2020	1416	612	0.98 (0.84, 1.13)	0.883	0.92 (0.81, 1.05)	0.722
Leptin	CRC risk by MSI status	MSI	2020	1416	96	0.96 (0.72, 1.28)	0.883	0.93 (0.71, 1.23)	0.722

a Hazard ratios (HRs) for CRC risk per 1 standard deviation (SD) increase in metabolic factors estimated with Cox regression, using age as a time scale, in complete case data and in the imputed data sets and aggregated using Rubin's rules.

Supplementary Table 3 CRC case characteristics by KRAS and BRAF data availability. Median (interquartile range) or n (%).

Variable	KRAS, BRAF or MSI status data missing (n=387)	All data available (n=623)	P[a]
**Sex, female (%)**	181 (47)	304 (49)	0.57
**BMI, kg/m2**	26.1 (19.0-43.6)	25.9 (17.4-46.7)	0.26
**Systolic blood pressure, mmHg**	130.0 (95.0-221.0)	134.0 (90.0-235.0)	0.4
**Diastolic blood pressure, mmHg**	82.0 (55.0-119.0)	85.0 (50.0-130.0)	0.26
**Glucose, mmol/l**	5.4 (2.7-12.7)	5.4 (2.3-13.3)	0.73
**Glucose tolerence, mmol/l**	6.5 (2.1-15.6)	6.7 (1.4-23.6)	0.04
**Total cholesterol, mmol/l**	6.0 (3.0-10.2)	6.0 (2.8-10.9)	0.62
**Triglycerides, mmol/l**	1.4 (0.4-6.1)	1.3 (0.4-15.2)	0.41
**Insulin, ng/mL**	0.2 (0.0-5.2)	0.2 (0.0-3.0)	0.77
**C-peptide, ng/mL**	1.3 (0.3-8.1)	1.3 (0.0-7.5)	0.76
**Adiponectin, mg/L**	19.0 (2.2-197.3)	19.7 (3.4-145.6)	0.22
**Leptin, ng/mL**	3.0 (0.1-52.5)	3.3 (0.1-142.5)	0.57
**Smoking, n (%)**			0.04
Never smoker	153 (40)	236 (39)	
Ex-smoker	122 (32)	237 (39)	
Current smoker	107 (28)	135 (22)	
**Occupational PA, n (%)**			0.42
1 (sedentary or standing work)	74 (22)	128 (25)	
2 (light but partly physically active)	70 (21)	108 (21)	
3 (light and physically active)	75 (23)	109 (22)	
4 (sometimes physically strenuous)	96 (29)	122 (24)	
5 (physically strenuous most of the time)	17 (5)	36 (7)	
**Recreational PA, n (%)**			0.08
1 (never)	141 (37)	279 (45)	
2 (every now and then – not regularly)	95 (25)	151 (24)	

3 (1-2 times/week)	88 (23)	109 (18)	
4 (2-3 times/week)	37 (10)	51 (8)	
5 (>3 times/week)	21 (5)	28 (5)	
**Age at diagnosis, years**	64.9 (39.5-83.9)	67.3 (37.8-89.6)	0.0002
**Year of diagnosis, n (%)**			0.1
1986-2006	151 (39)	203 (33)	
2007-2012	142 (37)	244 (39)	
2013-2016	94 (24)	176 (28)	
**Tumor site, n (%)**			0.003
Right colon	97 (26)	221 (36)	
Left colon	118 (31)	178 (29)	
Rectum	165 (43)	223 (36)	
**Tumor stage, n (%)**			0.25
Stage I&II	168 (48)	310 (52)	
Stage III&IV	182 (52)	285 (48)	

a P-values from Wilcoxon's tests of equal distributions in continuous variables or chi-square tests of equal distributions in categorical variables.

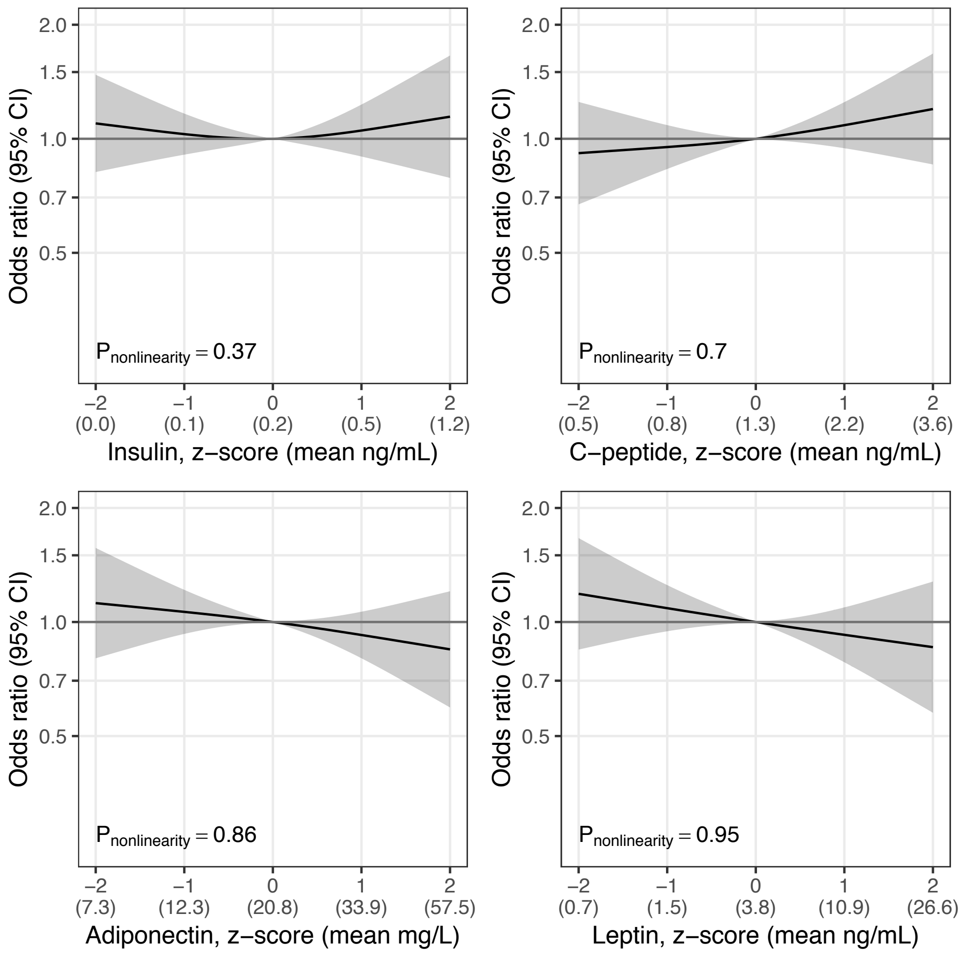

Supplementary Figure 1. Relative risks (RRs) of CRC by levels of metabolic biomarkers. Based on estimates from conditional logistic regression models, with biomarkers modeled with restricted cubic splines with knots at the 5th, 50th and 95th percentiles. Estimates were adjusted for matching variables and smoking, recreational and occupational physical activity, alcohol intake, and BMI. Values in parenthesis indicate mean non-transformed levels among participants with a z-score ±0.2 around the corresponding score. Nonlinearity was tested with a likelihood ratio test comparing the spline model to a linear model.

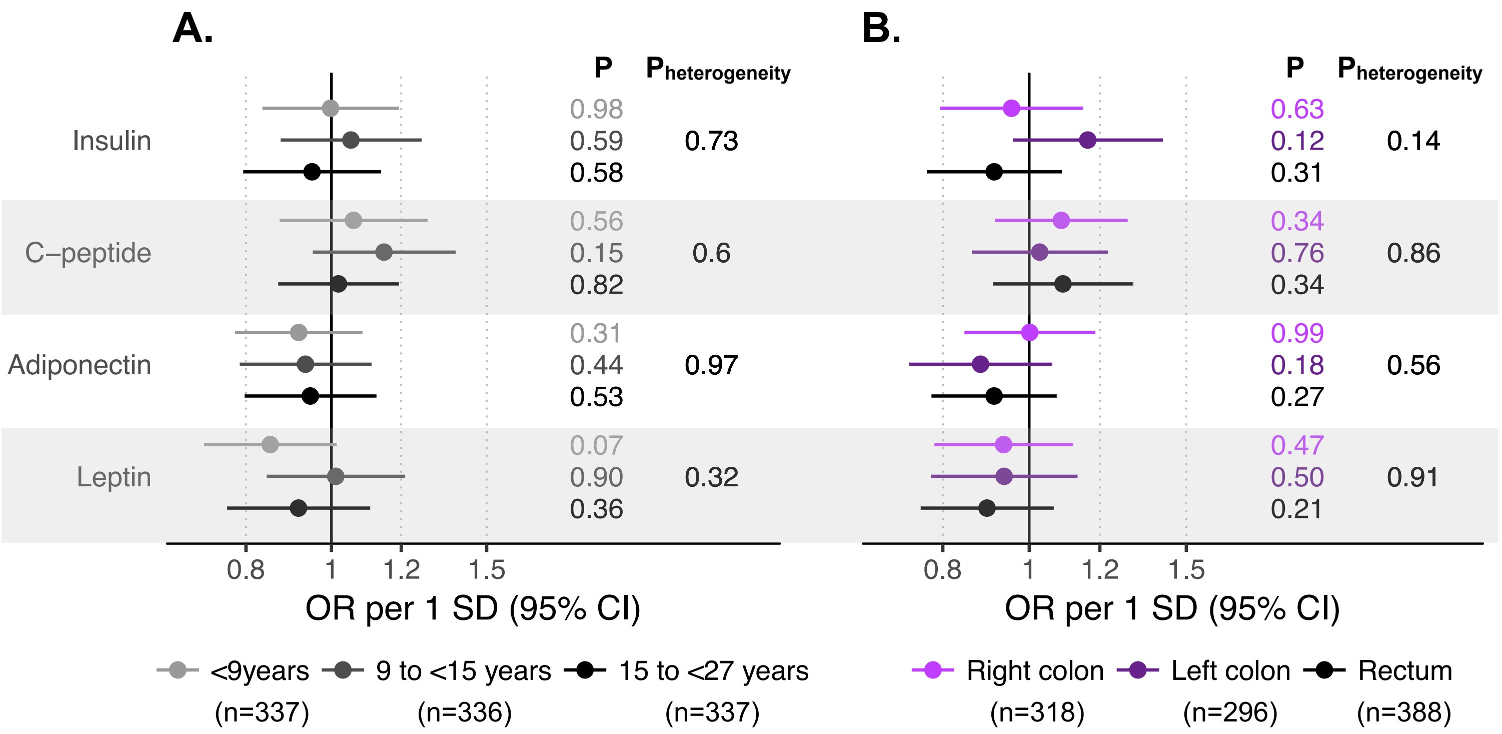

Supplementary Figure 2. Odds ratios (ORs) of CRC by (A) follow-up time between sampling and diagnosis and (B) tumor site per 1 SD increase in metabolic biomarkers. Estimated in 1010 cases and 1010 matched controls, adjusted for matching variables and smoking, occupational and recreational physical activity, alcohol intake, and BMI. Heterogeneity across tumor site was tested with a likelihood ratio test, comparing a model in which the risk association could vary across subtypes to a model were all associations were held constant. Numbers (n) within subtypes represent complete cases, data for the remaining cases were imputed.

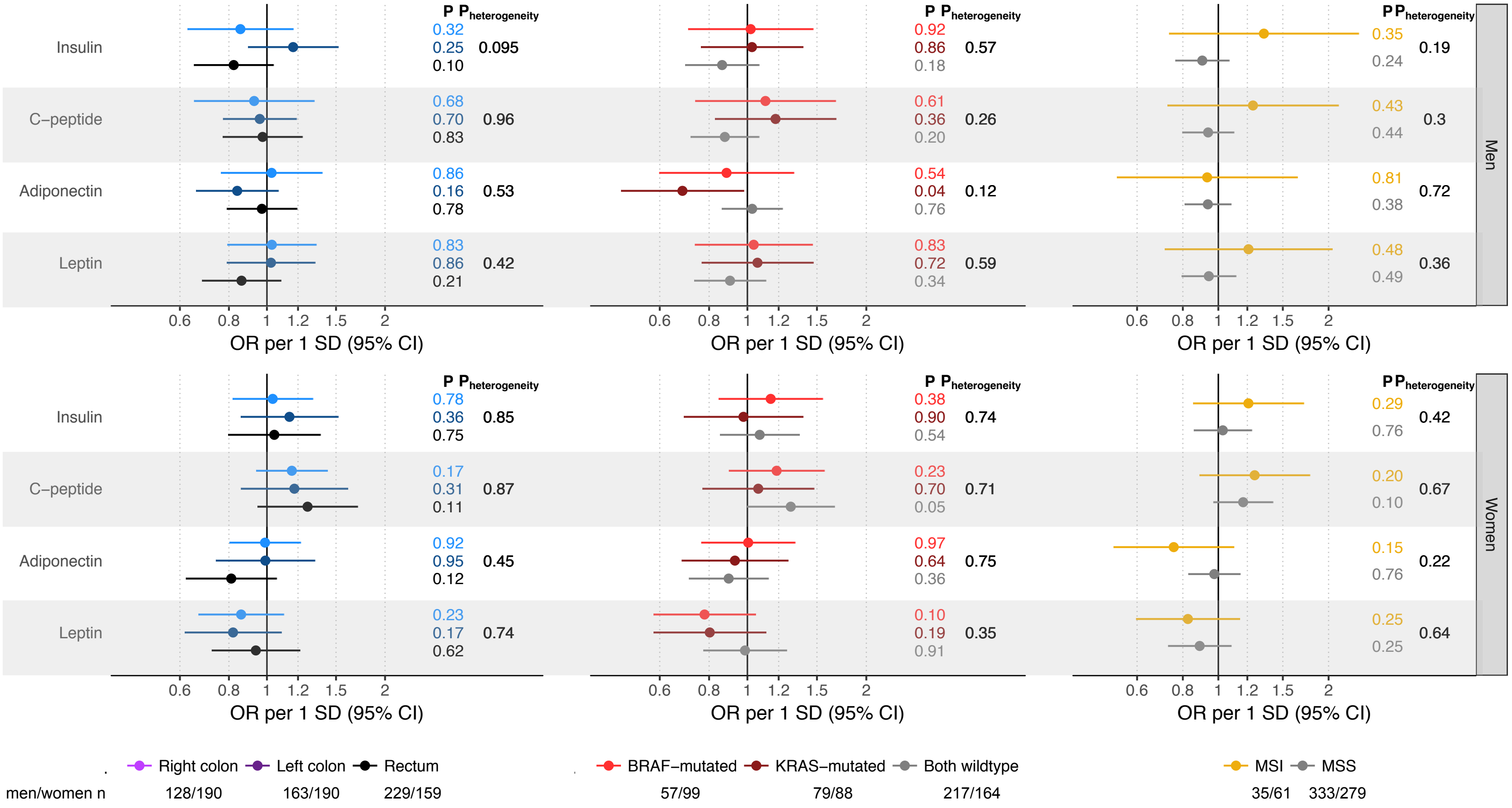

Supplementary Figure 3. Sex-specific odds ratios (ORs) of CRC by tumor site, *KRAS* and *BRAF* mutation status, and MSI status per 1 SD increase in metabolic biomarkers. Estimates were adjusted for matching variables and smoking, occupational and recreational physical activity, alcohol intake, and BMI. Heterogeneity across subtypes was tested with a likelihood ratio test, comparing a model in which the risk association could vary across subtypes to a model were all associations were held constant. Numbers (men/women) within subtypes represent complete cases, data for the remaining cases were imputed.
